# Astrocyte Proliferation Following Stroke in the Mouse Depends on Distance from the Infarct

**DOI:** 10.1371/journal.pone.0027881

**Published:** 2011-11-21

**Authors:** George E. Barreto, Xiaoyun Sun, Lijun Xu, Rona G. Giffard

**Affiliations:** 1 Department of Anesthesia, Stanford University School of Medicine, Stanford, California, United States of America; 2 Departamento de Nutrición y Bioquímica, Facultad de Ciencias, Pontificia Universidad Javeriana, Bogotá, Colombia; Boston University School of Medicine, United States of America

## Abstract

Reactive gliosis is a hallmark of brain pathology and the injury response, yet the extent to which astrocytes proliferate, and whether this is central to astrogliosis is still controversial. We determined the fraction of mature astrocytes that proliferate in a mouse stroke model using unbiased stereology as a function of distance from the infarct edge. Cumulatively 11.1±1.2% of Aldh1l1^+^ astrocytes within 400 µm in the cortical penumbra incorporate BrdU in the first week following stroke, while the overall number of astrocytes does not change. The number of astrocytes proliferating fell sharply with distance with more than half of all proliferating astrocytes found within 100 µm of the edge of the infarct. Despite extensive cell proliferation primarily of microglia and neutrophils/monocytes in the week following stroke, few mature astrocytes re-enter cell cycle, and these are concentrated close to the infarct boundary.

## Introduction

Astrogliosis is a hallmark of diseased CNS tissue [1]. This term refers to progressive changes in gene expression and cellular morphology, often including proliferation. However, data bearing on the extent and importance of astrocyte proliferation in reactive astrogliosis are inconsistent. Which gene expression changes are required, and whether astrocyte activation encompasses more than one activated phenotype, require further clarification. Reports both of little cell division and some estimates as high as 50% leave the contribution of astrocyte proliferation to reactive astrogliosis a subject of debate [2]–[4]. Here we used unbiased stereology to determine the extent of astrocyte proliferation as a function of distance from the infarct in the penumbra following stroke.

More studies on astrocyte proliferation have been published in brain stab wound injury than in stroke. Astrocytes accounted for up to one-fifth of the dividing cells in traumatic brain injury, but mitotic astrocytes represented only 2% of the total astrocyte population [5]–[8]. Similar proportions were estimated by Janeczko et al. [9], [10] and Schiffer et al. [11], [12] following brain injury. Several investigators have suggested that astrocyte proliferation contributes only a small number of cells [8], [13]–[18], and one quantitative study found no evidence of proliferation [19]. Despite the suggestion in some studies that there is little proliferation, studies with GFAP-TK mice suggest a robust proliferative astrocyte response, and that this proliferation is important to scar formation [3], [4]. Another study which identified a high rate of astrocyte proliferation was performed after stab wound injury and assessed cells within 150 µm of the injury track [2].

A further important question is whether proliferation occurs in immature cells or even progenitor cells which express GFAP in the adult brain [20], rather than in mature astrocytes. The number of astrocytes, as assessed by both glutamine synthetase (GS) and S100β immunoreactivity in the cortical penumbra following stroke does not increase strikingly over time, while GFAP and vimentin expression do [21]. To assess proliferation of mature astrocytes, compared to other cell types, we quantitated proliferation using BrdU labeling in Aldh1l1-GFP mice to identify mature cortical astrocytes [22] as well as double immunolabeling to identify other cell types.

## Materials and Methods

### Ethics Statement

All experiments with mice were performed according to an animal protocol (APLAC 9883) approved by the Stanford Animal Care and Use Committee, and in keeping with the National Institutes of Health guide.

### Mice

*Tg(Fthfd-EGFP)OFC789Gsat (Aldh1l1-GFP) mice*. Transgenic mice expressing EGFP from the Aldh1l1 locus in the Gsat BAC were initially produced by Nathaniel Heintz and colleagues [23] and were kindly provided by Ben Barres who obtained them from MMRRC (http://www.mmrrc.org/strains/11015/011015.html).

### Focal Cerebral Ischemia

Transient ischemia was induced using the suture occlusion technique, as previously described [24]. Adult male mice 25±5 g were anesthetized, and the left carotid artery bifurcation was exposed. A 6–0 monofilament nylon suture (Doccol Co., Redlands, CA, USA) was inserted from the common carotid artery into the internal carotid artery to occlude the left middle cerebral artery (MCA) at its origin. After 60 min the suture was removed for reperfusion, the CCA ligated, and the wound closed. Sham-operated animals underwent identical procedures as far as isolation of the carotid bifurcation but without opening the artery or suture insertion. Rectal temperature was maintained at 37°C±0.5°C controlled by a Homeothermic blanket control unit (Harvard Apparatus, Holliston, MA, USA). Temperature and respiratory rate were monitored during the surgery.

### Tissue Fixation and Immunohistochemistry

On day 3 and 7 after stroke or sham-operation mice were sacrificed for analysis. Animals were deeply anesthetized with isoflurane, and perfused through the left cardiac ventricle, first with 0.9% cold saline and then with 4% paraformaldehyde in phosphate-buffered saline (PBS; pH 7.4). Brains were removed and immersed overnight at 4°C in the same fixative and then rinsed with 0.1 M PBS. Coronal sections, 40 µm thick, were made with a Vibratome (VT 1000 S; Leica Microsystems, Wetzlar, Germany).

### Proliferation studies

Aldh1l1-GFP mice received three doses of BrdU (50 mg/kg in saline intraperitoneally; B5002, Sigma-Aldrich, St. Louis, MO) on day 2 and were sacrificed on day 3; or for cumulative proliferation, animals received three BrdU injections per day on days 2–6, and were sacrificed on day 7. Sections were processed for BrdU immunohistochemistry followed by double labeling with glial and neutrophil/monocytes markers. For BrdU immunostaining, sections were pretreated with 2 N HCL for 30 min at 37°C followed by 3 washes in 0.1 M PBS then incubated with polyclonal sheep anti-BrdU (1∶50, ab1893, Abcam, UK) for 2 days. Sections were then rinsed with 0.1 M PBS and incubated with Alexa 594 donkey anti-sheep (1∶500, Invitrogen) for 2 h. After 3 washes in PBS, sections were incubated with primary antibody for glial fibrillary acidic protein (GFAP, diluted 1∶5, #22522, Immunostar, Hudson, WI, USA), rabbit anti-S100β (diluted 1∶200, Z0311, DAKO, Carpinteria, CA), rat anti-CD68 (clone ED1, diluted 1∶200, MCA1957GA, Serotec, Raleigh, NC), rabbit anti-Iba1 (1∶400; 019–19741, Wako Chemicals USA, Richmond, VA) or rabbit anti-MPO (diluted 1∶200, A0398, DAKO, Carpenteria, CA). Sections were then incubated with respective secondary antibody, washed 3 times in 0.1 M PBS, mounted, and coverslipped with mounting medium (Vector Laboratories, CA). Representative micrographs were photographed at 20× or 40× using a Zeiss LSM 510 META inverted laser scanning confocal microscope (Zeiss, Germany). Z-stacks of 8 planes with 1 µm spacing were recorded in 6 min intervals. All counts were performed on coded sections so the investigator was blinded to the treatment group.

### Immunofluorescence for cleaved (active) caspase 3

For the detection of apoptosis in frozen tissue, sections were pretreated with 10 mM citrate buffer pH 6.0 at 80°C for 45 min. Sections were then cooled down for 15 min in the same buffer at room temperature (RT), washed 3 times in 0.1 M PBS and incubated with polyclonal rabbit anti-cleaved caspase 3 (diluted 1∶10, AB3623, Millipore, Bedford, MA) for 2 days. Sections were rinsed with 0.1 M PBS and incubated with Alexa 594 goat anti-rabbit (diluted 1∶500, Invitrogen) for 2 h at RT, mounted and coverslipped with mounting medium (Vector Laboratories).

### Morphometric analysis

**Figure 1:**
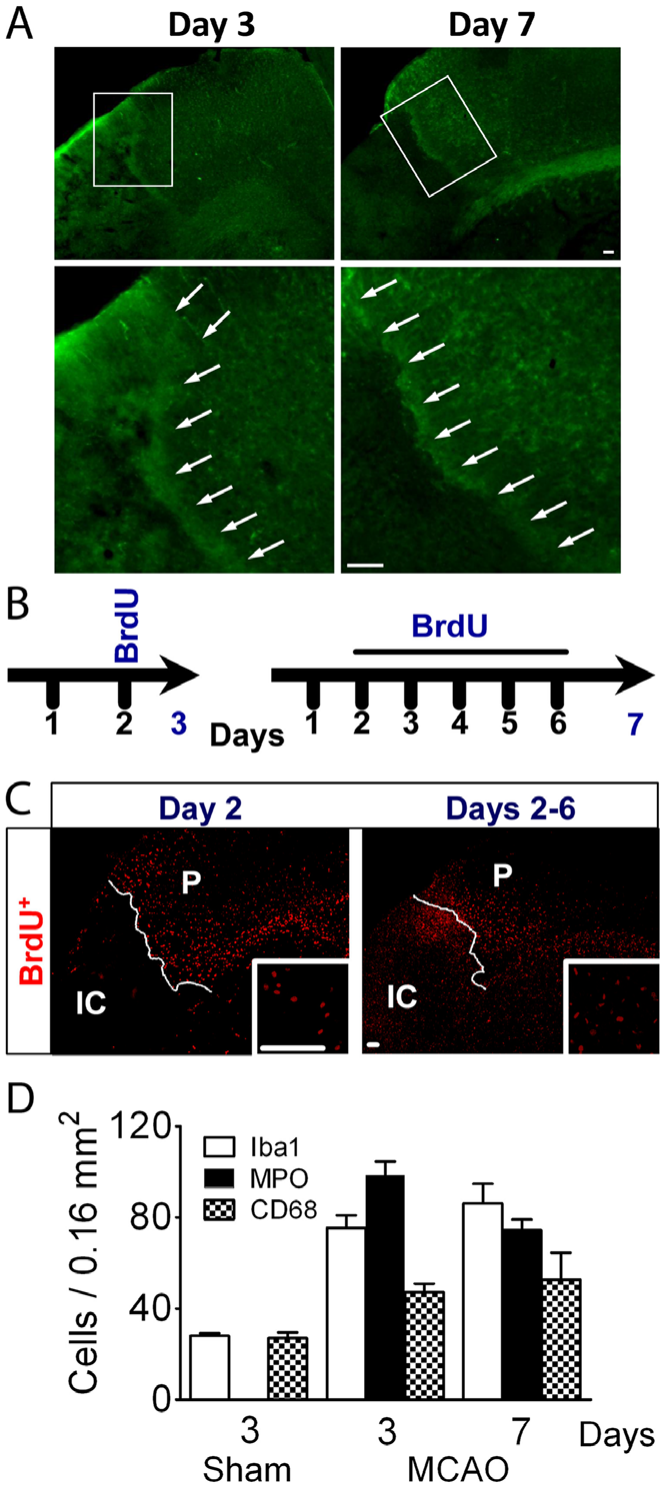
Cellular proliferation assessed with BrdU. (A) Low power micrographs show the edge of the core outlined by Aldh1l1 fluorescent astrocytes (green) present at the boundary visibly separating the core from the surrounding tissue on days 3 and 7 after ischemia; higher magnification of the areas indicated in the white boxes are shown below the low power images. (B) To study the proliferative response BrdU was injected 3 times/day on day 2 and animals were sacrificed on day 3 to determine the early response. A second group was injected 3 times/day from days 2–6 to analyze cumulative proliferation over the week following stroke, with animals sacrificed on day 7. (C) Panoramic views of sections immunostained for BrdU show proliferation in both penumbra (P) and ischemic core (IC), with somewhat stronger staining in penumbra on day 2, while the core showed more labelled cells on days 2–6. (D) Morphometric analysis shows the density of each cell type in the penumbra, demonstrating an increased inflammatory reaction surrounding the core. Sham animals were also analyzed for the number of each cell type on day 3 and 7, but the numbers for each cell type did not change significantly, so only the data for day 3 is shown. No MPO positive cells were detected in the sham animals. Scale Bar, 50 µm.

Brains that showed a similar infarcted area were used for morphometry to avoid the confound of differing extents of injury. The border between normal appearing cells and infarcted tissue is readily identified after 24 h [25] by the morphological appearance of nuclei. The core was identified as the region in which the majority of DAPI stained nuclei were shrunken, or the edge by appearance of Aldh1l1 fluorescent astrocytes (Figure 1A) whereas the penumbra was defined as the region of generally morphologically normal cells, 400–500 µm wide, surrounding the core. The number of Aldh1l1, GFAP, CD68, Iba1, MPO, or caspase 3 immunoreactive cells and double labelled cells immunoreactive for BrdU and cell type specific markers, was assessed in layer II-III of the cortical penumbra and within a distance of 400 µm from the border of the ischemic core of 3 mice for day 3 and 5 mice for day 7. Immunoreactive cells were estimated by the fractionator dissector method and a total of 16 counting frames of 100×100 µm each/section were counted. Three sections/animal (equally spaced between −1.70 to −2.18 mm relative to Bregma) were analyzed, comprising about 25% of the cortical penumbra using Image J software to analyze the micrographs.

### Statistics

Data are expressed as mean ± SEM. The differences between groups were determined by *t-*test when only two groups were compared; differences between multiple groups were determined by one way analysis of variance followed by *post hoc* Tukey's multiple comparison test, using Graphpad Prism 5 (GraphPAD Software for Science, San Diego, CA, USA). Differences were considered statistically significant for *p*-values<0.05.

## Results

**Figure 2:**
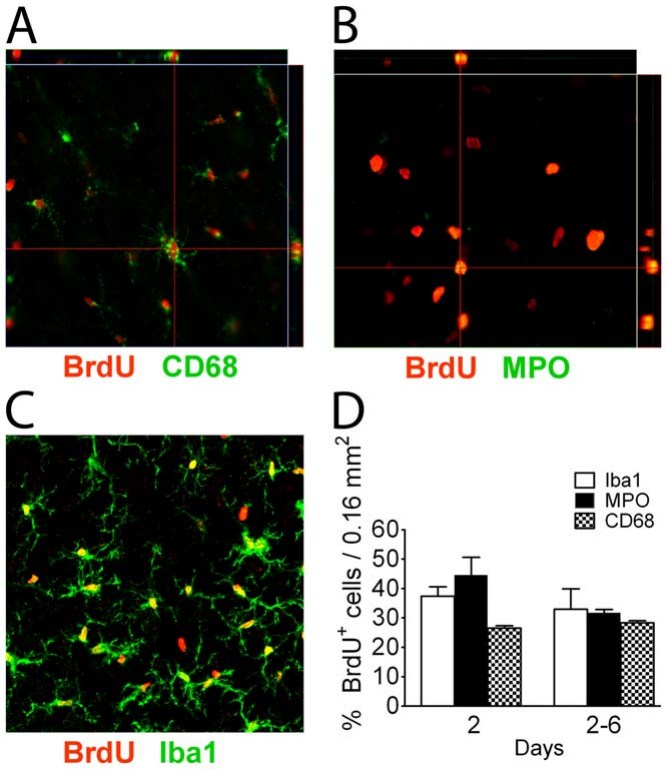
Analysis of proliferating cells in the penumbra identifies many inflammatory cells. Using different antibodies to identify the cell types labelled with BrdU, reveals that most proliferating cells are microglia/macrophages (A, C) and monocytes/neutrophils by colocalization (B) of cell type markers with BrdU, and this was confirmed by the morphometric analysis (D). N = 3 animals for day 2, N = 5 animals for day 7. Scale Bar, 50 µm.

Serial confocal pictures were taken in the border area of the ischemic penumbra/ischemic core for unbiased stereological image analysis to quantitate the response of Aldh1l1^+^ astrocytes to injury. Early proliferation was assessed by injecting BrdU on day 2, and cumulative proliferation was determined by repetitive BrdU injections from day 2–6 after MCAO (Figure 1B). Proliferation is greater in the cortical penumbra on day 2, with little BrdU labelling in the ischemic core (Figure 1C). By day 6 post MCAO, this changes to pronounced proliferation in the core (Figure 1C) with some ongoing proliferation in the penumbra (Figures 1C,D). Cell counting was performed within 400 µm of the IC/P border, but beyond the glial scar which develops at the IC/P boundary. Proliferating cells consisted predominantly of morphologically large and small round cells. The number of MPO^+^ cells rises dramatically from undetectable levels in the sham condition, and CD68^+^ and Iba1^+^ cells were present at high density in the penumbra on day 3 (Figure 1D). Representative low power micrographs of immunostaining for Iba1, MPO, and CD68 in the penumbra on days 3 and 7 are shown in Figure S2. The increased number of activated microglia and monocytes on day 7, relative to day 3, demonstrates an increased immunological and inflammatory response bordering the lesion (Figure 1D). Double labeling for BrdU and myeloperoxidase (MPO), a marker for polymorphonuclear neutrophils and monocytes, Iba1 for resting and activated microglia/macrophages, and CD68 for activated microglia/macrophages (Figures 2A–C) demonstrated that the large round proliferating cells were MPO^+^ neutrophils/monocytes, and accounted for 44.5±6.1% and 31.7±1.1% of BrdU^+^ cells on days 2 and 2–6, respectively. The small proliferating cells were microglia/macrophages, corresponding to 37.4±3.1% and 32.9.0±7.5% for Iba1^+^ immunoreactive cells, 26.5±0.7% and 28.3±0.6% for CD68^+^ cells, on days 2 and 2–6, respectively (Figure 2D).

**Figure 3:**
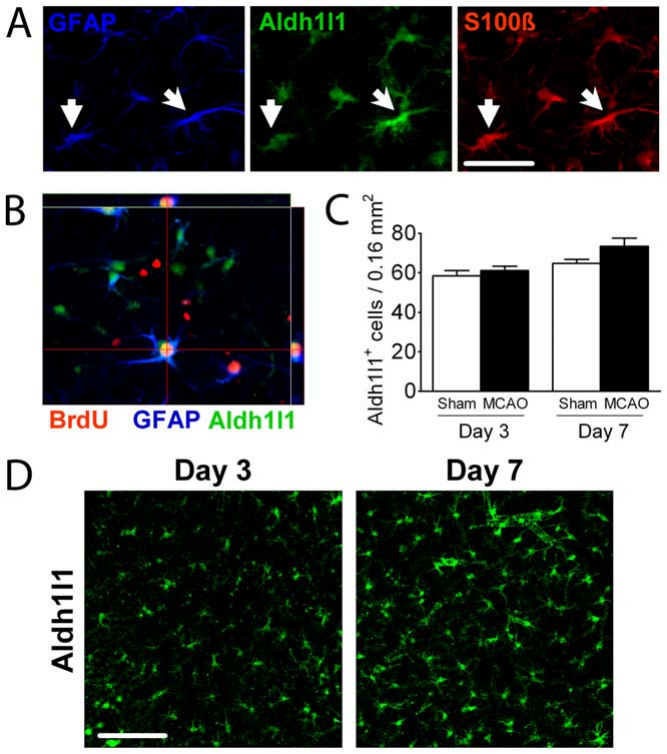
Few Aldh1l1^+^ astrocytes proliferate in the cortical penumbra. (A) Triple labelling reveals that most astrocytes in the cortical penumbra label for GFAP, Aldh1l1 and S100β. (B) Orthogonal view of a mature astrocyte co-expressing Aldh1l1, BrdU and GFAP in the cortical penumbra. (C) The number of Aldh1l1^+^ astrocytes assessed in layers II–III of the cortical penumbra does not change over the first week post-injury, or compared to sham animals. (D) Illustrative confocal micrographs show increased prominence of Aldh1l1 positive cells with cellular hypertrophy, but no change in overall number. Scale Bar, 50 µm.

Aldh1l1^+^ astrocytes co-express GFAP (53.9±2.0% on day 3 within 400 um of the core) and S100β in the penumbra (Figure 3A and Figure S1), and orthogonal views show that some GFAP/Aldh1l1 double positive cells also label with BrdU (Figure 3B). The total number of Aldh1l1^+^ astrocytes was not significantly different on post-stroke day 3 or 7 compared to sham (Figures 3C,D). This was consistent with the small fraction of Aldh1l1 positive cells that labeled for BrdU^+^ (8.5±1.2% for day 2; 11.1±1.2% cumulatively for days 2–6), and by the small percentage of BrdU^+^ cells that were also positive for Aldh1l1 (6.4±1.4% for day 2; 8.7±0.6% for days 2–6), suggesting that mature astrocytes do not proliferate extensively after stroke, and that proliferation happens early.

**Figure 4:**
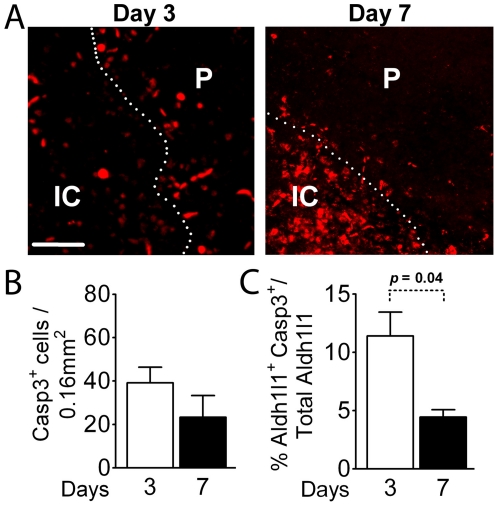
Detection of apoptotic cells in the penumbra. (A) Apoptotic cells identified by staining for cleaved caspase 3 show few apoptotic cells in the core and some in the penumbra on day 3, contrasting with a striking increase in apoptotic cells in the core on day 7. (B) Quantitation of apoptotic cells in the penumbra on days 3 and 7 after injury. (C) A small percent of Aldh1l1 cells are also apoptotic as assessed by caspase 3 activation. The number significantly decreases between days 3 and 7. IC = ischemic core; P = penumbra. N = 3 mice for day 2, N = 5 mice for day 7. Scale Bar, 50 µm.

The finding that about 10% of Aldh1l1^+^ astrocytes incorporate BrdU raises the question of whether this might be an underestimate due to loss of astrocytes by apoptosis. To address this question we stained sections for cleaved caspase 3 to assess the number of apoptotic Aldh1l1^+^ astrocytes on days 3 and 7 post MCAO. A few apoptotic cells are found in the core on post-stroke day 3, contrasting with greater numbers of caspase 3 immunoreactive cells in the penumbra, while on day 7 there continue to be large numbers of apoptotic cells in the core, but few in the penumbra (Figures 4A–C). The morphometric analysis shows that 11.4±2.1% of Aldh1l1^+^ cells co-label with caspase 3 on day 3, and 4.4±0.6% on day 7 (Figure 4C) thus accounting for the overall lack of change in numbers of Aldh1l1 cells, with apoptosis like proliferation occurring early after the injury.

**Figure 5:**
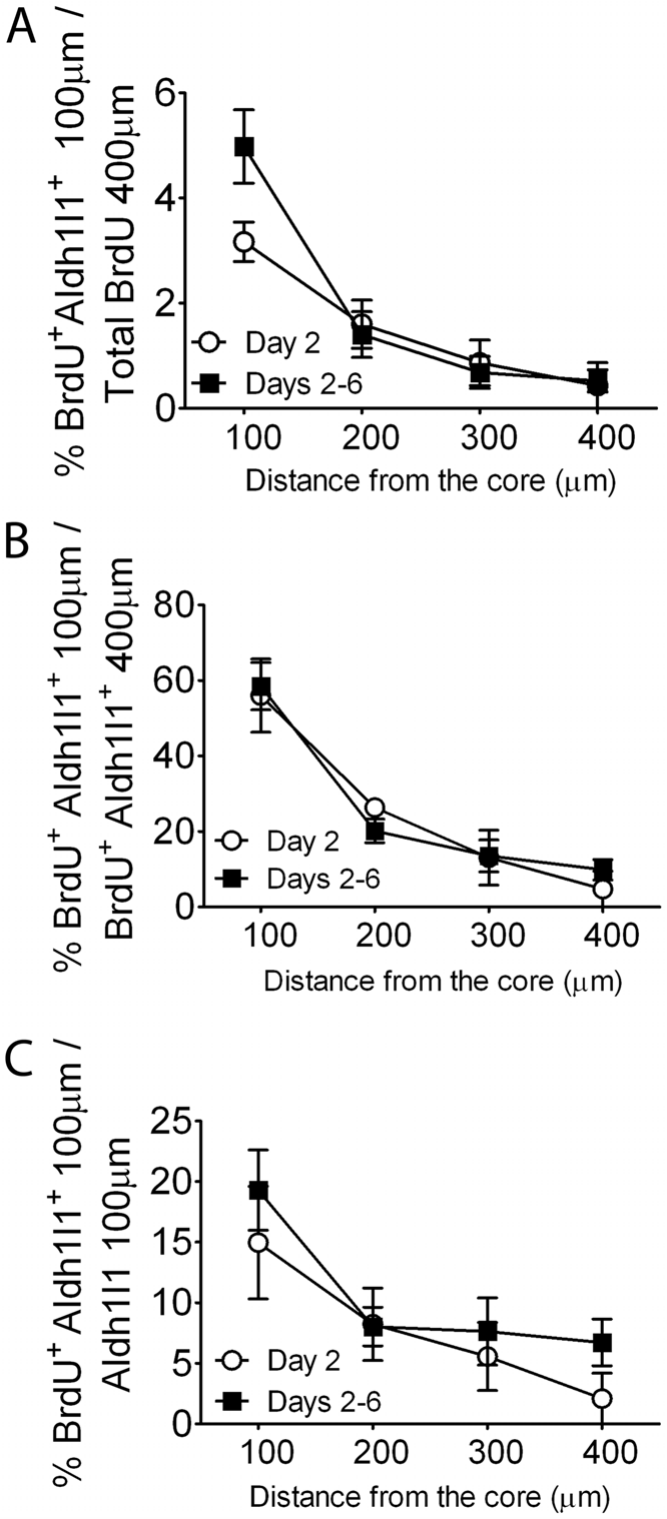
Astrocyte proliferation as a function of distance from the infarct core. BrdU labelling on day 2 or 2–6 cumulatively shows a small percentage of mature Aldh1l1^+^ astrocytes proliferate after MCAO within 400 µm of the ischemic core. Stereological analysis of astrocyte proliferation as a function of distance shows that Aldh1l1^+^ cells mainly co-express BrdU in the first 200 µm from the infarct area; the number decreases dramatically with distance. The fraction of proliferating astrocytes compared to all proliferating cells in the penumbra is low (A), and the fraction of proliferating astrocytes present falls rapidly with distance (B), and the fraction of all astrocytes present that proliferate is highest close to the core (C).

One hypothesis that could reconcile the divergent estimates of astrocyte proliferation following injury is the concept that the probability of astrocytes reentering the cell cycle is a function of their distance from the injury. Because our stereological study used 100 µm square counting frames we were able to analyze the number of BrdU^+^ Aldh1l1^+^ cells as a function of distance from the edge of the infarct. When BrdU^+^ Aldh1l1^+^ cells are analyzed as a fraction of all BrdU^+^ cells the fraction falls rapidly with distance from the core (Figure 5A). The percent of Aldh1l1^+^ BrdU^+^ cells found in each 100 µm compared to the total number of Aldh1l1^+^ BrdU^+^ cells also fell rapidly with distance (Figure 5B), with about 60% of all BrdU labeled astrocytes within 100 µm of the core, and about 80% within 200 µm. The maximum percent of Aldh1l1 cells that labeled was about 20% (Figure 5C) and this was seen within 100 µm of the scar. Similar patterns are observed with labeling on day 2 or days 2–6.

## Discussion

Proliferation of mature astrocytes after stroke in the cortical penumbra cumulatively over the first week was surprisingly modest at 11.1%. This data reinforces the concept that mature astrocytes do not widely resume proliferation after stroke. Astrocytes at different distances from the edge of the ischemic core undergo graded responses to injury, including graded degrees of proliferation. There is clear evidence from others for astrocyte proliferation at the scar boundary, while astrocytes further out show reactive changes, but as shown quantitatively in this study, largely without proliferation. Our analysis of proliferation with distance from the injury allows us to reconcile several previously disparate observations on the extent of astrocyte proliferation.

The highest levels of astrocytes proliferation have been observed either in the scar or close to the edge of a stab wound [26], [27]. Using cell counting methods, Alonso [26] showed that GFAP^+^ cells at a distance of 200–250 µm from the wound accounted for ∼20% and ∼27% of all BrdU labelled cells on days 3 and 7, respectively, following stab wound. More recently, Buffo et al. [2] reported up to 50% of fate marked astrocytes within 150 µm of the wound labelled with BrdU. This relatively high fraction is consistent with proliferation largely restricted to the scar and immediately next to the scar. In stroke there is a prior report of extensive GFAP^+^ astrocyte proliferation close to the infarct area in the rat [20] but no unbiased quantitation was provided.

In contrast several other reports demonstrate low levels of astrocyte proliferation after injury. A different study in stroke in the rat determined that 10% of GFAP astrocytes within 250 µm of the core co-labeled with BrdU in the cortex [28], quite consistent with our observations. Low levels of proliferation were also found in a study in human ischemic stroke [29]. Using a genetic fate-mapping and BrdU-labeling approach Burns et al. [30] showed that cortical astrocytes cease proliferation before P10 and only undergo nonproliferative gliosis (i.e., increased GFAP expression without cell-division) after stab-wound injury in adult brains, though they detected proliferation of NG2 positive cells. Similarly, Dihne et al. [31] concluded that the unchanged density of astrocytes in striatum in the days following excitotoxic CNS injury suggests increased GFAP-reactivity without proliferation.

We observe the highest level of proliferation closest to the edge of the infarct, which falls rapidly within 200 µm. The average exclusive territory or domain size occupied by a single hippocampal astrocyte is in the range of 24,000–66,000 µm^3^ [32], [33], and similar observations about the domain structure of astrocytes have been made in cortex [34]. This corresponds to a linear dimension in the range of 50 to >100 µm for the domain of a single astrocyte, suggesting that the astrocytes that undergo proliferation either have the infarct directly within their territory, or border on it. This fits nicely with earlier observations that there is significant proliferation of astrocytes involved in scar formation [2], [4], [27] and suggests that the extent to which astrocytes experience destruction of their exclusive territory or domain is a key determinant of whether they undergo proliferation. It allows us to reconcile some of the widely different estimates of astrocyte proliferation in the literature based on the distance from the injury where the assessment was performed. Our results fit with a broader understanding of the graded response of astrocytes to injury. This encompasses proliferation to form the scar, an intermediate activation phenotype in which some astrocytes may undergo proliferation and in which there may be limited disruption of domain structure, and finally a more distal activated astrocyte population in which there is no proliferation, no loss of domain structure, and activation is indicated only by changes in gene expression including increased expression of GFAP and vimentin [4].

A smaller percentage of astrocytes positive for Aldh1l1 undergo apoptosis on day 7 than on day 3 after injury, with the overall extent similar to that observed for proliferation, and consistent with the overall lack of change in numbers of astrocytes. Other cell types, namely microglia, monocytes, and progenitors, have been shown to undergo apoptosis after stroke [35] and readily account for the non-astrocytic apoptotic cells seen.

The findings reported here provide detailed quantitation of cell proliferation following focal ischemia in the mouse, as a function of distance from the scar. It is likely that the range of astrocyte response to other injuries also falls along the continuum discussed here between scar forming astrocytes exhibiting relatively high levels of proliferation to astrocytes that do not proliferate at all, but do show gene expression and morphological changes indicative of activation. Thus following stroke, astrocytes in the penumbra undergo limited proliferation and apoptosis, soon after injury, with a strong spatial gradient. Most astrocyte proliferation occurs within 200 µm of the edge of the infarct.

## Supporting Information

Figure S1 **A higher power view of the border on day 7 shows astrocytes extending their processes into the edge of the core, with extensive but not complete colocalization of Aldh1l1^+^ (green channel) with GFAP (blue channel) and S100β (red channel).** Scale bar, 50 µm. GFAP and S100β identified by immunostaining.
(TIF)

Figure S2 **Low power micrographs showing representative staining for Iba 1, MPO, and CD68 in the penumbra on days 3 and 7.** Scale bar, 50 µm.
(TIF)

## References

[1] Pekny M, Nilsson M (2005). Astrocyte activation and reactive gliosis.. Glia.

[2] Buffo A, Rite I, Tripathi P, Lepier A, Colak D (2008). Origin and progeny of reactive gliosis: A source of multipotent cells in the injured brain.. Proc Natl Acad Sci U S A.

[3] Sofroniew MV (2009). Molecular dissection of reactive astrogliosis and glial scar formation.. Trends Neurosci.

[4] Sofroniew MV, Vinters HV (2010). Astrocytes: biology and pathology.. Acta Neuropathol.

[5] Cavanagh JB (1970). The proliferation of astrocytes around a needle wound in the rat brain.. J Anat.

[6] Latov N, Nilaver G, Zimmerman EA, Johnson WG, Silverman AJ (1979). Fibrillary astrocytes proliferate in response to brain injury: a study combining immunoperoxidase technique for glial fibrillary acidic protein and radioautography of tritiated thymidine.. Dev Biol.

[7] Ludwin SK (1985). Reaction of oligodendrocytes and astrocytes to trauma and implantation. A combined autoradiographic and immunohistochemical study.. Lab Invest.

[8] Reier PJ, Fedoroff S, Vernadakis A (1986). Gliosis following CNS injury, the anatomy of astrocytic scars and their influences on axonal elongation.. Astrocytes.

[9] Janeczko K (1988). The proliferative response of astrocytes to injury in neonatal rat brain. A combined immunocytochemical and autoradiographic study.. Brain Res.

[10] Janeczko K (1989). Spatiotemporal patterns of the astroglial proliferation in rat brain injured at the postmitotic stage of postnatal development: a combined immunocytochemical and autoradiographic study.. Brain Res.

[11] Schiffer D, Giordana MT, Cavalla P, Vigliani MC, Attanasio A (1993). Immunohistochemistry of glial reaction after injury in the rat: double stainings and markers of cell proliferation.. Int J Dev Neurosci.

[12] Schiffer D, Giordana MT, Migheli A, Giaccone G, Pezzotta S (1986). Glial fibrillary acidic protein and vimentin in the experimental glial reaction of the rat brain.. Brain Res.

[13] Fernaud-Espinosa I, Nieto-Sampedro M, Bovolenta P (1993). Differential activation of microglia and astrocytes in aniso- and isomorphic gliotic tissue.. Glia.

[14] Hajos F, Gerics B, Turai E (1993). Astroglial reaction following Wallerian degeneration in the rat visual cortex: proliferation or hypertrophy?. Neurobiology (Bp).

[15] Hatten ME, Liem RK, Shelanski ML, Mason CA (1991). Astroglia in CNS injury.. Glia.

[16] Lindsay RM, Fedoroff S, Vernadakis A (1986). Reactive gliosis.. Astrocytes.

[17] Ridet JL, Malhotra SK, Privat A, Gage FH (1997). Reactive astrocytes: cellular and molecular cues to biological function.. Trends Neurosci.

[18] Vaughn JE, Pease DC (1970). Electron microscopic studies of wallerian degeneration in rat optic nerves. II. Astrocytes, oligodendrocytes and adventitial cells.. J Comp Neurol.

[19] Norenberg MD (1994). Astrocyte responses to CNS injury.. J Neuropathol Exp Neurol.

[20] Liu Y, Namba T, Liu J, Suzuki R, Shioda S (2010). Glial fibrillary acidic protein-expressing neural progenitors give rise to immature neurons via early intermediate progenitors expressing both glial fibrillary acidic protein and neuronal markers in the adult hippocampus.. Neuroscience.

[21] Petito CK, Morgello S, Felix JC, Lesser ML (1990). The two patterns of reactive astrocytosis in postischemic rat brain.. J Cereb Blood Flow Metab.

[22] Cahoy JD, Emery B, Kaushal A, Foo LC, Zamanian JL (2008). A transcriptome database for astrocytes, neurons, and oligodendrocytes: a new resource for understanding brain development and function.. J Neurosci.

[23] Gong S, Zheng C, Doughty ML, Losos K, Didkovsky N (2003). A gene expression atlas of the central nervous system based on bacterial artificial chromosomes.. Nature.

[24] Han RQ, Ouyang YB, Xu L, Agrawal R, Patterson AJ (2009). Postischemic brain injury is attenuated in mice lacking the beta2-adrenergic receptor.. Anesth Analg.

[25] Dingman A, Lee SY, Derugin N, Wendland MF, Vexler ZS (2006). Aminoguanidine inhibits caspase-3 and calpain activation without affecting microglial activation following neonatal transient cerebral ischemia.. Journal of Neurochemistry.

[26] Alonso G (2005). NG2 proteoglycan-expressing cells of the adult rat brain: possible involvement in the formation of glial scar astrocytes following stab wound.. Glia.

[27] Myer DJ, Gurkoff GG, Lee SM, Hovda DA, Sofroniew MV (2006). Essential protective roles of reactive astrocytes in traumatic brain injury.. Brain.

[28] Popa-Wagner A, Badan I, Walker L, Groppa S, Patrana N (2007). Accelerated infarct development, cytogenesis and apoptosis following transient cerebral ischemia in aged rats.. Acta Neuropathol.

[29] Dziewulska D (1997). Age-dependent changes in astroglial reactivity in human ischemic stroke. Immunohistochemical study.. Folia Neuropathol.

[30] Burns KA, Murphy B, Danzer SC, Kuan CY (2009). Developmental and post-injury cortical gliogenesis: a genetic fate-mapping study with Nestin-CreER mice.. Glia.

[31] Dihne M, Block F, Korr H, Topper R (2001). Time course of glial proliferation and glial apoptosis following excitotoxic CNS injury.. Brain Res.

[32] Bushong EA, Martone ME, Jones YZ, Ellisman MH (2002). Protoplasmic astrocytes in CA1 stratum radiatum occupy separate anatomical domains.. J Neurosci.

[33] Ogata K, Kosaka T (2002). Structural and quantitative analysis of astrocytes in the mouse hippocampus.. Neuroscience.

[34] Halassa MM, Fellin T, Takano H, Dong JH, Haydon PG (2007). Synaptic islands defined by the territory of a single astrocyte.. J Neurosci.

[35] Broughton BR, Reutens DC, Sobey CG (2009). Apoptotic mechanisms after cerebral ischemia.. Stroke.

