# Supplementary figures and images for: Astrocyte Proliferation Following Stroke in the Mouse Depends on Distance from the Infarct
Source: PLoS One. 2011 Nov 21;6(11):e27881. doi: 10.1371/journal.pone.0027881 (PMC3221692; doi:10.1371/journal.pone.0027881)

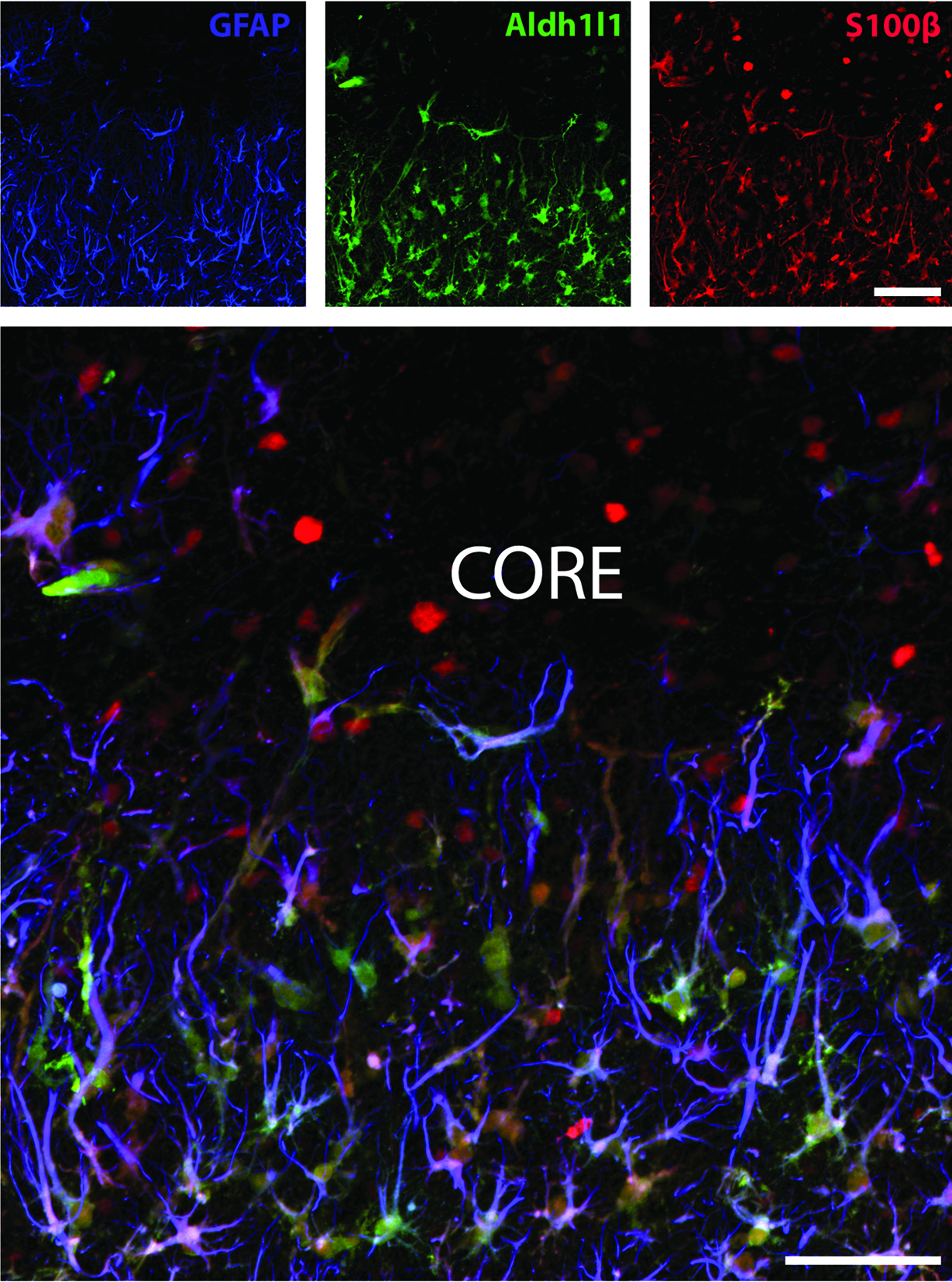

Supplement: Figure S1 — A higher power view of the border on day 7 shows astrocytes extending their processes into the edge of the core, with extensive but not complete colocalization of Aldh1l1+ (green channel) with GFAP (blue channel) and S100β (red channel). Scale bar, 50 µm. GFAP and S100β identified by immunostaining. (TIF) [file pone.0027881.s001.tif]

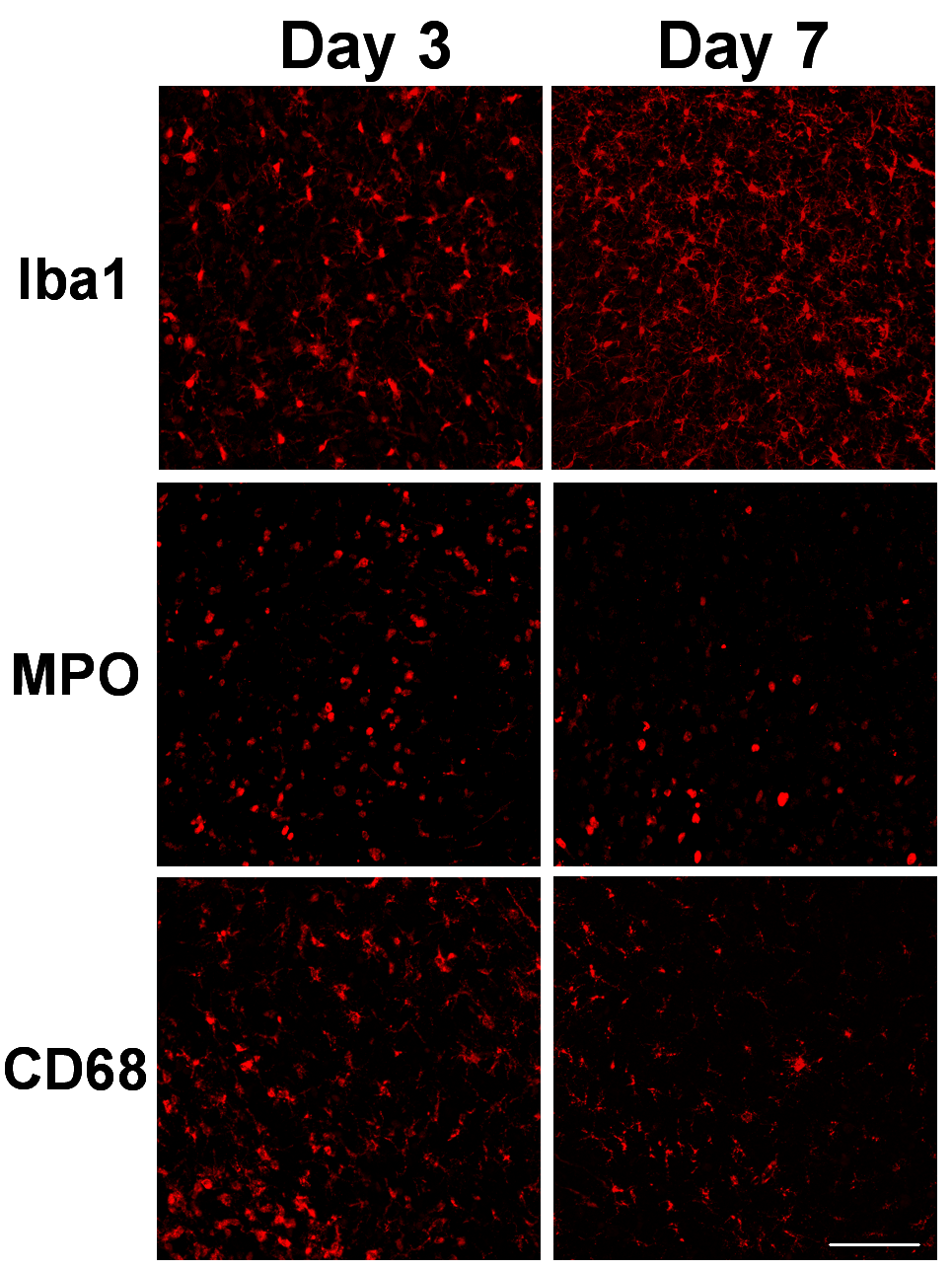

Supplement: Figure S2 — Low power micrographs showing representative staining for Iba 1, MPO, and CD68 in the penumbra on days 3 and 7. Scale bar, 50 µm. (TIF) [file pone.0027881.s002.tif]
